# Laparoscopic Repair of Morgagni Hernia: Three-Case Presentation and the Literature

**DOI:** 10.1155/2016/4268539

**Published:** 2016-11-10

**Authors:** Gholamali Godazandeh, Farzad Mokhtari-esbuie

**Affiliations:** ^1^Department of Thoracic Surgery, School of Medicine, Mazandaran University of Medical Sciences, Sari, Iran; ^2^Department of Surgery, Emam Khomeyni Hospital, Mazandaran University of Medical Sciences, Sari, Mazandaran, Iran

## Abstract

*Introduction*. Morgagni hernia is a rare form of congenital diaphragmatic hernia.* Case Presentation*. We present three cases of Morgagni hernia with GI symptoms treated by laparoscopic surgery.* Discussion*. Hernial sac was excised in two cases and left in situ in one case. There was no recurrence in symptoms after 30 months from surgery.

## 1. Introduction

Congenital diaphragmatic hernias (CDH) are rare congenital defects. There are some different types; Bochdalek hernia, Morgagni hernia, and esophageal hiatus hernia, described with case presentations. Bochdalek hernia is the most common type of congenital diaphragmatic hernia. Anteromedial or Morgagni hernia is the least common variety, accounting for only 1–3% of all diaphragmatic hernias [1–3]. It is caused by a defect in the retrosternal region of the diaphragm and is considered to occur due to failure of fusion in the anterior part of the pleuroperitoneal membrane and deficiency in the process of muscularization [4].

Morgagni hernia is congenital. However, there were some patients that had previous normal radiography suggesting that these hernias may be acquired through a congenital diaphragmatic defect [5].

Morgagni hernia is more common on the right side, at the level of the seventh rib on either side of the xiphoid, in a space where the superior epigastric vessels pass; defects may also occur on the left, at the midline, or bilaterally; that on the left side is referred to as Larrey hernia [6].

From one-third to more than half of patients are asymptomatic. These patients may be found incidentally when a chest X-ray undertaken for investigating unrelated problems [7].

If symptoms are present, the symptoms and signs are usually related to the size of the sac and contents in the hernial sac. The most symptoms present in these cases include nausea, vomit, recurrent chest infection, and chest pain [3, 4, 8].

Complications such as intermittent gastric volvulus, small intestine obstruction, incarceration, and strangulation of the hernia could occur. So surgical treatment is indicated in all cases of CDH even in asymptomatic patients to prevent complication [4, 9, 10].

We present three cases of Morgagni hernia repaired by laparoscopy.

## 2. Case Presentation

### 2.1. Case  1

A 75-year-old woman with vague epigastric pain one year earlier was admitted to our hospital. Her epigastric pain worsened with large solid meals. She also suffer from nausea, vomiting, and constipation. Symptoms worsened since one month prior to admission. No sweating, palpitation, dyspnea, anorexia, weight loss, dysphagia, and odynophagia were detected. A chest X-ray (CXR) revealed the presence of a mass in the proximity of the right lung.

### 2.2. Case  2

**Figure 1:**
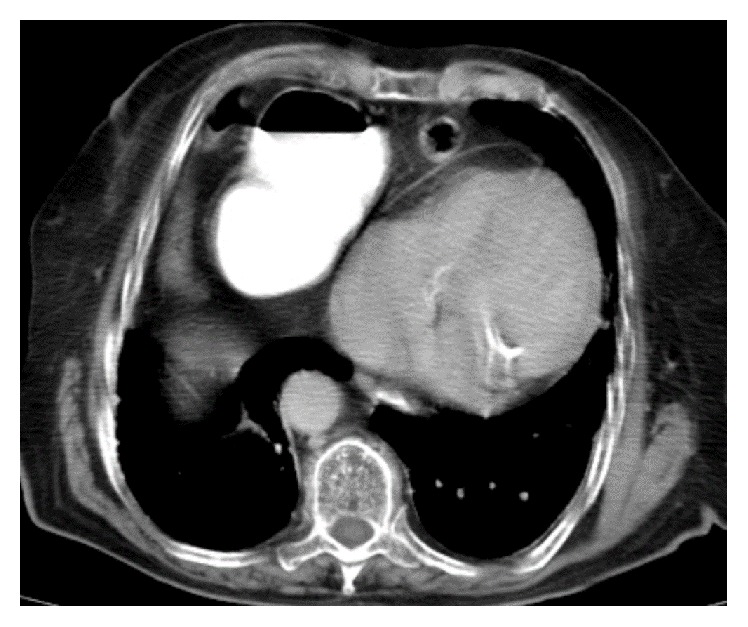
Thoracic CT scan. Stomach filled by contrast in right hemithorax.

An 80-year-old woman came to our hospital with chief compliant of cough, dyspnea at rest, and orthopnea since two weeks before admission. She also had vomiting after heavy meals, generalized vague abdominal pain, dysphagia, regurgitation, epigastric fullness, constipation, and sweating. Clinical exam and diagnostic imaging including CXR and spiral thoracoabdominal CT scan were compatible with Morgagni hernia (Figure 1).

### 2.3. Case  3

**Figure 2:**
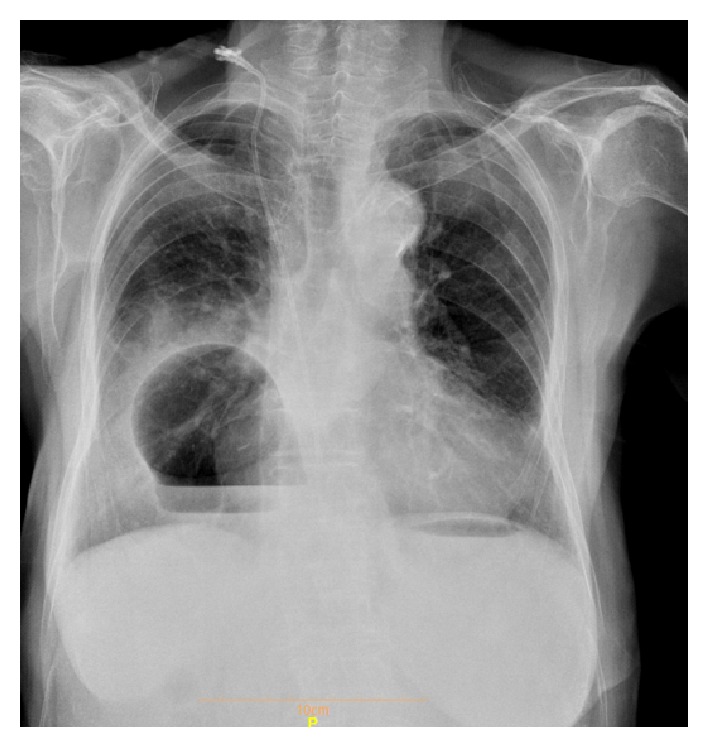
Chest X-ray. Mass with air-fluid level in right hemithorax.

An 82-year-old woman came to our hospital with chief complaint of nausea and vomiting that worsened after taking meal from 2 weeks ago. She had no epigastric pain or dysphagia or odynophagia and no weight loss was detected. She had no history of blunt trauma prior to this problem. A chest X-ray was done and showed round opacity with air-fluid level at right hemithorax on the dome of the diaphragm (Figure 2). Biochemical marker was normal. On further workup, thoracic CT scan was performed and revealed part of the colon, omentum, and stomach herniated into the thorax. When the diagnosis is confirmed, the patient was prepared for laparoscopic surgery.

After preoperative risk assessments including ECG, echocardiography, cardiologist consult, routine laboratory tests, and pulmonary function test, patients were prepared for surgical repair of the Morgagni hernia.

**Figure 3:**
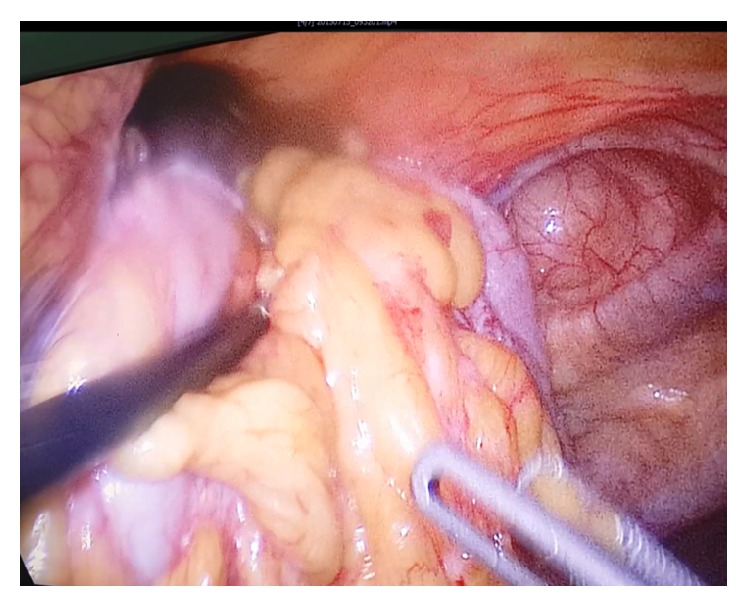
Intraperitoneal cavity. Pulling out hernia sac content back into abdomen by noncrashing grasper.

**Figure 4:**
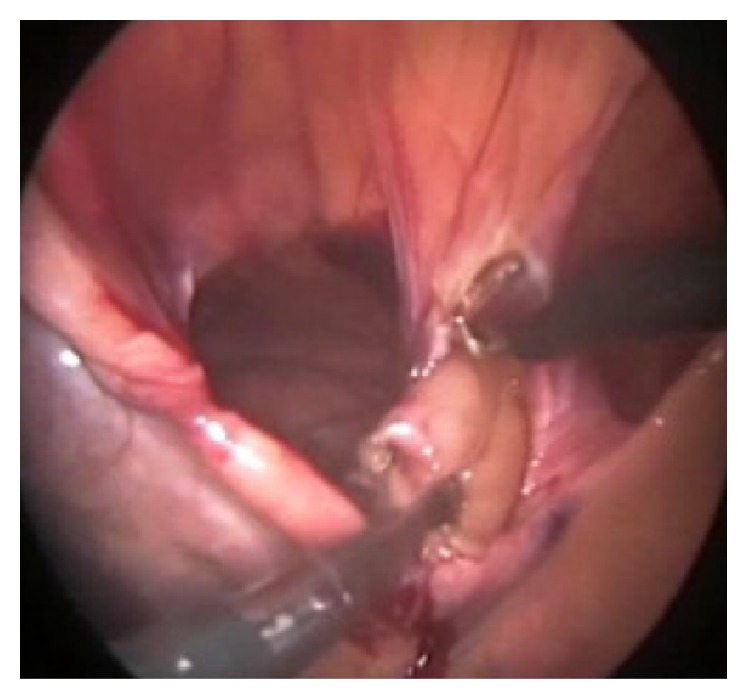
Excision of the sac.

**Figure 5:**
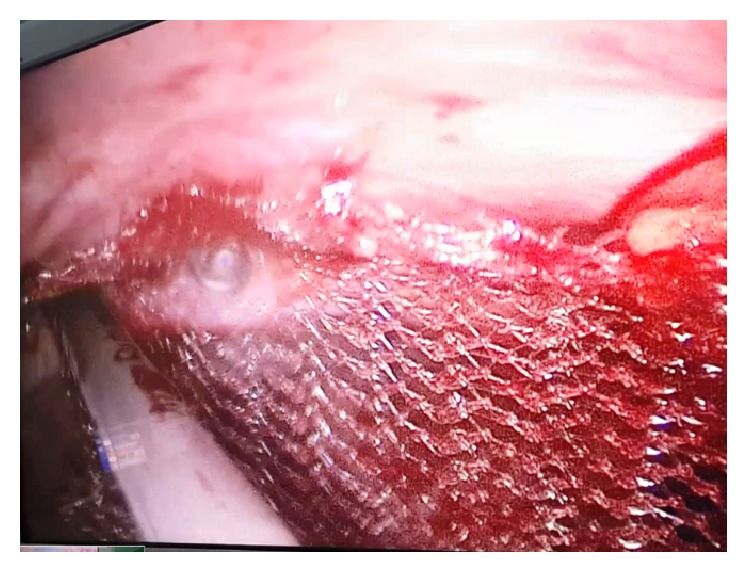
Fixation of mesh to anterior abdominal wall.

Patients were placed in the supine position. A Foley catheter was inserted after general anesthesia. Pneumoperitoneum with carbonic dioxide (CO_2_) was performed by Veress needle. One 10 mm trocar port was inserted above the umbilicus and a camera (30° angulated optic) was inserted into the abdominal cavity. Two additional trocars (5 mm) were placed at the right and the left of the abdomen, and one 10 mm trocar was placed at the left flank, respectively. Subsequently, patients were putted on the reverse Trendelenburg positions. The anesthesiologist manually inflated the lungs to ensure positive pressure in order to facilitate the reduction of the sac contents. Intraoperatively, hernial sac content (colon, omentum, and part of stomach) was pulled out and reduced back into the abdominal cavity (Figure 3). The falciform ligament was divided and the hernia sac was excised at first two cases by LigaSure (Figure 4), but at operation of the third patient, the hernial sac had not been removed. The size of the defect was 6 × 7 cm in the first patient, 5 × 6 cm in the second patient, and 6 × 6 cm in the third patient. Dual-sided mesh (15 × 20 cm) was inserted into the abdominal cavity through the 10 mm port in all three cases. Mesh was expanded over the defect and fixed to the anterior abdominal wall and edge of the diaphragmatic defect in the posterior part with spiral tacks (Protack®, Covidien, Mansfield, MA, USA) (Figure 5). After removal of the trocars under direct visualization, the fascial incision at the 10 mm trocar sites was closed via sutures. All patients were admitted to the SICU for the first postoperative day and discharged within 48 to 72 hours after surgery. All three cases were discharged ambulatorily and putted on normal diets. There was no complication such pneumomediastinum, fluid collection, or recurrence during 20 to 30 months of follow-up for all patient.

## 3. Discussion

Morgagni hernia was first described by Giovanni Battista Morgagni, an Italian anatomist and pathologist in 1769, while performing a postmortem examination on a patient who died of a head injury [11, 12].

Also the etiology of CDH is unknown; however, 2% of cases have been intended to be familial and another 15% of patients have associated chromosomal abnormality. It is more common in female and obese patients. All three patients of our experience were female.

Morgagni hernias usually have a sac. It is reported that the peritoneal sac of almost all Morgagni hernias was well developed and herniated into the thoracic cavity; and the most common parts herniated into thorax are colon, omentum, stomach, liver, or other viscera [13, 14].

The most present symptoms in these cases include nausea, vomit, recurrent chest infection, and chest pain [3, 4, 8].

Cardiopulmonary symptoms such as dyspnea and palpitations are infrequent and occur less frequently than GI symptoms.

Our cases present with different symptoms. Case 1 presented with nausea and vomiting while case 2 and case 3 presented with chest pain, cough, and dyspnea, respectively.

Differential diagnosis of this condition includes pleuropericardial cyst, pleural mesothelioma, pericardial fat pad, mediastinal lipoma, tumor or cyst of the diaphragm, thymoma, and anterior chest wall tumors [2].

The diagnosis is based on further imaging in the form of a barium study, computed tomography (CT), or magnetic resonance imaging (MRI). These kinds of imaging can define the size of the defect and contents of the hernial sac [4].

In our cases, CT scan was done for all three patients to define the diagnosis and exclude the other pathologies.

Morgagni hernia should be treated, even in asymptomatic cases. The surgery is not urgent except there is evidence of strangulation [5, 9].

Morgagni hernia can be repaired by a variety of surgical approaches including laparotomy, thoracotomy, laparoscopy, and thoracoscopy [4, 5, 15, 16].

An open transabdominal approach (laparotomy by upper midline or paramedian or subcostal incision) is the method of choice in patients with obstruction, incarceration, strangulation, or perforation. Both sides can be evaluated by a midline incision [9].

Transthoracic approach provides an excellent view for repair of the hernia. Most surgeons do not favor repair of MH by a thoracotomy due to its associated morbidity and need for chest tube drainage. Although thoracoscopic approach is a less invasive treatment, evaluation of the other side is not possible [17].

The laparoscopic approach was firstly reported by Kuster et al. in 1992 [18]. Laparoscopic repair is a safe, minimally invasive, and effective procedure and has been mentioned as the gold standard and the initial step for repair of a noncomplicated Morgagni hernia [8, 9]. Excellent bilateral view, less tissue damage, less requirement for postoperative analgesia, a short hospital stay, and rapid return to normal life are the stated benefits of laparoscopic repair for Morgagni hernia [3, 8, 19, 20].

Recently, various laparoscopic techniques have been described for repair of MH, which include a primary closure of the defect with intracorporeal sutures, stapler, or a mesh [4, 5].

However, there are some controversies regarding important aspects in the laparoscopic repair [7]. Morgagni hernias usually has a sac. It is reported that the peritoneal sac of almost all Morgagni hernias was well developed and herniated into the thoracic cavity. Excision of the hernial sac in MH remains a controversial issue. Some authors recommend excising hernial sac [4, 20, 21], whereas others prefer to leave the hernial sac in situ [22–24].

Excision of the sac may have the following advantages: (1) reduction of tissue trauma because only the sac is manipulated (rather than its contents) in cases where the colon or stomach is contained within the sac; (2) decreased chance for symptomatic fluid collection since the serous lining membrane is removed; and (3) sac excision that negates the chance that the sac itself can act as a lead point for recurrent herniation [21].

In contrast, there have been concerns against removal of the sac because it may result in massive pneumomediastinum, damage to the pericardium, and mediastinal structures which are life threatening [18].

In our cases, we resected hernial sac in case 1 and case 2. There was no complication during or after surgery on follow-up. We did not remove hernia sac in case 3, but no complication such fluid collection or recurrence occur after 20 months.

## 4. In Conclusion

 Morgagni hernia must be a differential diagnosis of persistent GI symptom. Our experience and the review of the literature indicate that laparoscopic repair of the Morgagni hernia is a safe approach. Hernial sac can be excised or left in situ.
